# Supplementary material for: 5- and 6‑Membered Rings: A Natural Orbital Functional Study
Source: J Chem Theory Comput. 2026 Mar 6;22(6):2799–807. doi: 10.1021/acs.jctc.5c01861 (PMC13019622; doi:10.1021/acs.jctc.5c01861)
Supplement: Supplementary file 1 [file ct5c01861_si_001.pdf]

Supporting Information for: "5- and 6-membered rings: A natural orbital functional study"

Ion Mitxelena,[*,†] Juan Felipe Huan Lew-Yee,[*,‡,¶,§] and Mario Piris[*,‡,∥,⊥]

†*Fisika Aplikatuko departamentua, Vitoria-Gasteiz Ingenieritza Eskola, Euskal Herriko Unibertsitatea (EHU), 01006 Vitoria-Gasteiz, Euskadi, Spain*

‡*Donostia International Physics Center (DIPC), 20018 Donostia, Euskadi, Spain*

¶*Departamento de Física y Química Teórica, Facultad de Química, Universidad Nacional Autónoma de México, México City, C.P. 04510, México*

§*Departamento de Matemáticas, Universidad Nacional Autónoma de México, México City, C.P. 04510, México*

∥*Polimero eta Material Aurreratuak: Fisika Kimika eta Teknologia, Euskal Herriko Unibertsitatea (EHU), 20018 Donostia, Euskadi, Spain*

⊥*Basque Foundation for Science (IKERBASQUE), 48009 Bilbao, Euskadi, Spain*

E-mail: ion.mitxelena@ehu.eus; felipe.lew.yee@dipc.org; mario.piris@ehu.eus

1 Calculations with the Dunning's cc-pVNZ basis set

In the present section, we show correlation energies obtained for the correlation-consistent Dunning's basis sets (cc-pVXZ, X=2-5). The latter are preferable to be used in calculations with frozen core electrons, however, due to their relevance to the historical development of NOF approximations and their widespread use, we include the corresponding correlation energy analysis below.

Correlation energies for GNOF, GNOFm, and CCSD(T) are shown in Fig. S1 for increas-

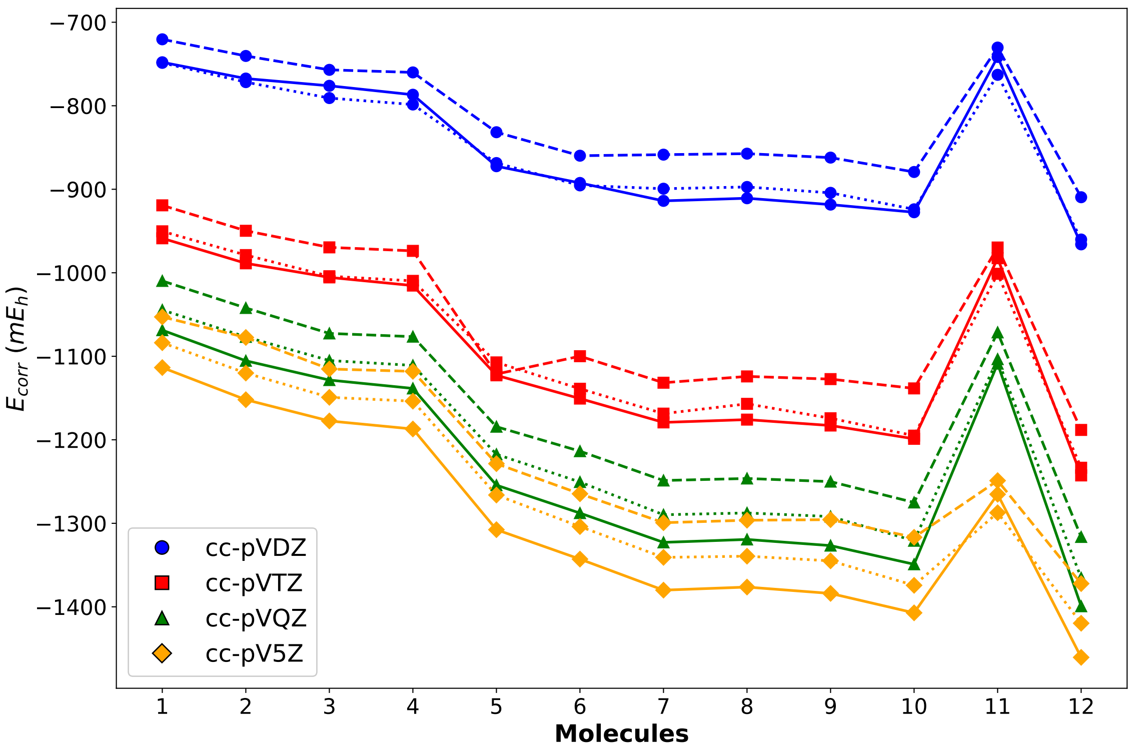

Figure S1: Correlation energies ($E - E_{\mathrm{HF}}$) in mE_h for the selected set of molecules, obtained by using GNOF (dashed lines), GNOFm (dotted lines), and CCSD(T) (solid lines) using the cc-pVXZ basis sets. The cardinal numbers X = 2, 3, 4, and 5 correspond to blue, red, green and orange, respectively. Molecules ordered according to the numbering given in Table S1.

ing size cc-pVXZ basis sets, being X=2-5 the cardinal number of the basis. Here, molecules are ordered according to the numbering presented in Table S1. NOF and CCSD(T) curves are roughly parallel, so the molecular description agrees for both methods independently of the size of the basis set, as well as of the different studied molecular rings.

GNOF and GNOFm complete basis set limit (CBS) correlation energy estimates are given in Table S1, which also presents CCSD(T) values, performed using the same procedure. Helgaker's extrapolation scheme, $E_\infty + bX^{-3}$ with $X = 2, 3, 4, 5$, was employed to carry out the CBS extrapolation. An inspection of CBS limit molecular correlation energies reveals an agreement within 100 mE_h between GNOF and CCSD(T), values that are even improved to around 50 mE_h when GNOFm is utilized. The results shown in Table S1 are summarized in Fig. S2. GNOFm energies systematically get closer to CCSD(T) results when the static

Table S1: Complete basis set (CBS) extrapolated correlation energies ($E - E_{\mathrm{HF}}$) in mE_h for the 12 molecular systems, computed using GNOF, GNOFm, and CCSD(T). Helgaker's extrapolation scheme, $E_\infty + bX^{-3}$, was employed with $X = 2, 3, 4, 5$ as the cardinal number of the basis set.

No.	**Systems**	**GNOF**	**GNOFm**	**CCSD(T)**
1	Cyclopentadiene	-1051.3	-1084.4	-1110.6
2	Pyrrole	-1081.7	-1118.9	-1149.0
3	Furan	-1114.7	-1148.2	-1173.7
4	Imidazole	-1118.3	-1153.1	-1183.4
5	Benzene	-1245.5	-1265.8	-1304.0
6	Pyridine	-1262.8	-1301.7	-1338.9
7	Pyrazine	-1302.9	-1343.0	-1375.2
8	Pyrimidine	-1298.5	-1338.3	-1371.6
9	Pyridazine	-1299.9	-1346.6	-1379.0
10	Triazine	-1320.3	-1374.8	-1402.0
11	Thiophene	-1179.6	-1214.3	-1204.5
12	Tetrazine	-1372.4	-1419.3	-1453.8

term between electron pairs is modified as described in the section II from the manuscript. In other words, Fig. S2 reveals that GNOFm CBS correlation energies reduce differences between GNOF and CCSD(T) to the half. Additionally, as it is shown in Fig. S1, the improvement is obtained for all basis sets studied.

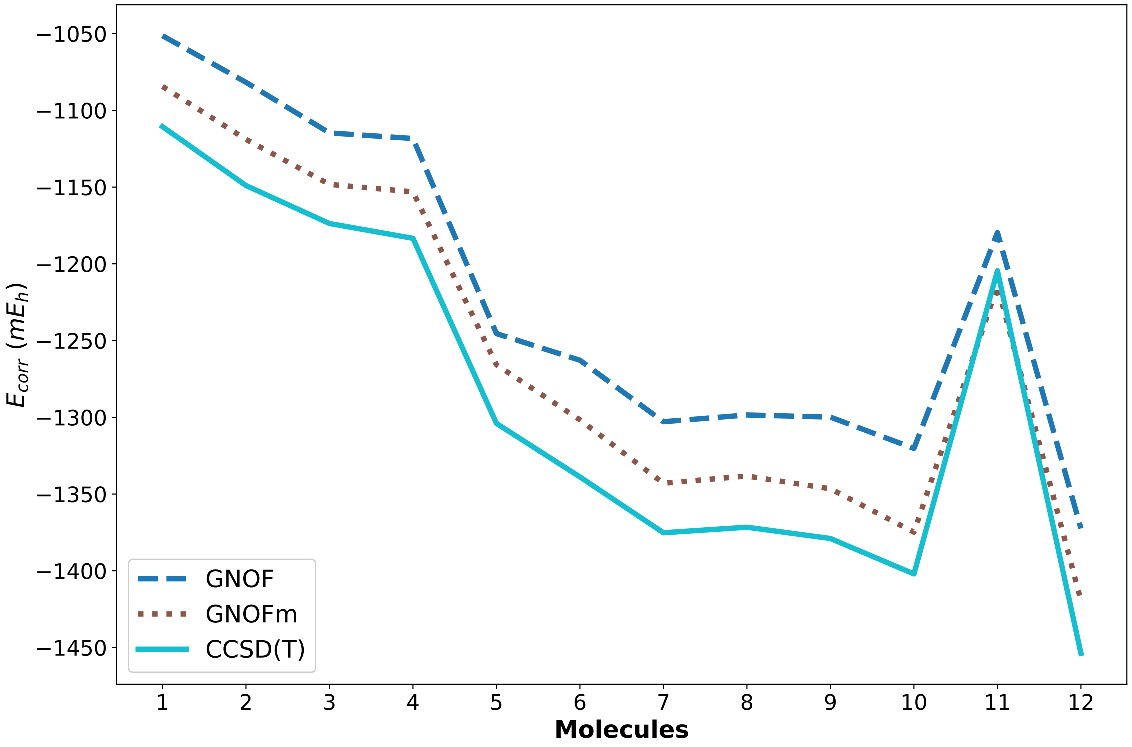

Figure S2: Complete basis set (CBS) extrapolated correlation energies ($E - E_{\mathrm{HF}}$) in mE_h for the 12 molecular systems, computed using GNOF, GNOFm, and CCSD(T). Helgaker's extrapolation scheme, $E_\infty + bX^{-3}$, was employed with $X = 2, 3, 4, 5$ as the cardinal number of the basis set.

2 Molecular dipole moments

In this section, we show the dipole moments obtained by using GNOF and GNOFm approximations, together with reference Hartree-Fock and experimental values. Literature sources corresponding to the latter, as well as numerical values (in a.u.), are given in Table S2. Both GNOF and GNOFm improves Hartree-Fock values and corresponding dipole moments get closer to experimental data when the basis set is increased beyond cc-pwCVDZ, as it

is shown for Pyrrole, Imidazole, or Pyridazine. GNOF provides the most accurate values for the largest cc-pwCVQZ basis set, whereas GNOFm dipole moments are similar for the latter basis sets. Thiophene molecule corresponds to largest errors when the largest basis set is employed, and the latter do not improve if d functions are removed from the basis set. In fact, corresponding dipole moments for the cc-pwCVQZ basis set read as 0.2703 a.u. and 0.2833 a.u., respectively for GNOF and GNOFm, which do not change significantly the errors obtained with the aug-cc-pwCVQZ basis set as it is shown in Table S2.

Table S2: Non-zero dipole moments (in a.u.) computed using HF, GNOF and GNOFm with Dunning's cc-pwCVXZ basis sets ($X = 2, 3, 4$), together with experimental values and their corresponding reference from the literature. Results corresponding to thiophene (no. 11) are obtained with the aug-cc-pwCVXZ basis sets. Molecular numbering reads as follows: 1. Cyclopentadiene, 2. Pyrrole, 3. Furan, 4. Imidazole, 6. Pyridine, 8. Pyrimidine, 9. Pyridazine, 11. Thiophene.

**Systems**	**1**	**2**	**3**	**4**	**6**	**8**	**9**	**11**
HF/cc-pwCVDZ	0.143	0.778	0.298	1.505	0.871	0.906	1.689	0.269
HF/cc-pwCVTZ	0.143	0.755	0.299	1.509	0.895	0.930	1.707	0.294
HF/cc-pwCVQZ	0.148	0.745	0.308	1.512	0.907	0.941	1.722	0.298
GNOF/cc-pwCVDZ	0.160	0.776	0.270	1.480	0.851	0.904	1.639	0.258
GNOF/cc-pwCVTZ	0.159	0.737	0.283	1.475	0.870	0.910	1.655	0.266
GNOF/cc-pwCVQZ	0.165	0.727	0.291	1.476	0.885	0.913	1.663	0.271
GNOFm/cc-pwCVDZ	0.163	0.762	0.271	1.470	0.847	0.893	1.627	0.283
GNOFm/cc-pwCVTZ	0.159	0.731	0.282	1.463	0.856	0.895	1.646	0.270
GNOFm/cc-pwCVQZ	0.167	0.724	0.286	1.462	0.886	0.904	1.656	0.271
**Exp.**	**0.165**	**0.695**	**0.268**	**1.444**	**0.871**	**0.918**	**1.660**	**0.216**
**Ref.**	(1)	(2)	(3)	(4)	(5)	(6)	(7)	(8)

References

(1) Laurie, V. W. Microwave Spectrum and Dipole Moment of Cyclopentadiene. *J. Chem. Phys.* **1956**, *24*, 635–636.

(2) Nygaard, U.; Nielsen, J.; Kirchheiner, J.; Maltesen, G.; Rastrup-Andersen, J.; Sørensen, G. Microwave spectra of isotopic pyrroles. Molecular structure, dipole moment, and 14N quadrupole coupling constants of pyrrole. *J. Mol. Struct.* **1969**, *3*, 491–506.

(3) Khakoo, M. A.; Muse, J.; Ralphs, K.; da Costa, R. F.; Bettega, M. H. F.; Lima, M. A. P. Low-energy elastic electron scattering from furan. *Phys. Rev. A* **2010**, *81*, 062716.

(4) Christen, D.; Griffiths, J. H.; Sheridan, J. The Microwave Spectrum of Imidazole; Complete Structure and the Electron Distribution from Nuclear Quadrupole Coupling Tensors and Dipole Moment Orientation. *Z. Naturforsch. A* **1981**, *36*, 1378–1385.

(5) Sørensen, G. O.; Mahler, L.; Rastrup-Andersen, N. Microwave spectra of [15n] and [13c] pyridines, quadrupole coupling constants, dipole moment and molecular structure of pyridine. *J. Mol. Struct.* **1974**, *20*, 119–126.

(6) Blackman, G.; Brown, R.; Burden, F. The microwave spectrum, dipole moment, and nuclear quadrupole coupling constants of pyrimidine. *J. Mol. Struct.* **1970**, *35*, 444–454.

(7) Battaglia, M. R.; Ritchie, G. L. D. Kerr constants, Cotton–Mouton constants, and magnetic anisotropies of pyridazine, pyrimidine, and pyrazine. *J. Chem. Soc., Perkin Trans. 2* **1977**, 897–900.

(8) A contribution to the structure determination of Ar–thiophene: the electric dipole moment. *Chem. Phys.* **1998**, *239*, 199–206.
